# External Validation of the Diagnosis of TIA (DOT) Score for Identification of TIA in a Chinese Population

**DOI:** 10.3389/fneur.2019.00796

**Published:** 2019-08-13

**Authors:** Junliang Yuan, Zejin Jia, Yangguang Song, Wenli Hu

**Affiliations:** ^1^Department of Neurology, Beijing Chaoyang Hospital, Capital Medical University, Beijing, China; ^2^Department of Pathology, Beijing Chaoyang Hospital, Capital Medical University, Beijing, China

**Keywords:** transient ischemic attack, diagnosis of TIA score, Dawson score, ischemic stroke, diffusion weighted imaging

## Abstract

**Background:** Recently, the [diagnosis of transient ischemic attack (TIA), DOT] score has been recognized to be a new tool for non-specialists to diagnose TIA more accurately with the sensitivity and specificity being 89 and 76%, respectively. However, the DOT score has not yet been validated externally in patients with TIA in China.

**Methods:** We retrospectively enrolled 500 consecutive patients with transient neurological symptoms, who were admitted to the Department of Neurology, Beijing Chaoyang Hospital and underwent magnetic resonance imaging (MRI) between Jan 2016 and Dec 2018. Patients with transient neurological symptoms were divided into two subgroups: TIA mimic group (*N* = 140, 28%) and definite cerebrovascular events group including tissue-based TIA (DWI negative, *N* = 252, 50.4%) and minor stroke (DWI positive, *N* = 108, 21.6%). The demographic data, clinical characteristics, laboratory findings, and scores of Dawson and DOT were compared between the two groups.

**Results:** A total of 500 patients with transient neurological symptoms (mean age, 61.1 ± 12.8) were enrolled and 70% (*N* = 350) were male. Comparing with TIA mimic groups, patients with cerebrovascular events group were more likely to have higher diastolic blood pressure, uric acid and homocysteine, more motor weakness and speech abnormalities, and also scored higher using the Dawson and DOT. The area under the curve (AUC) was 0.728 for DOT, with a sensitivity of 70.3% and specificity of 62.9%, respectively.

**Conclusion:** In patients with transient neurological symptoms, our findings showed that the DOT score had relatively good calibration and discrimination to identify of TIA in a Chinese Population. As a novel tool of TIA identification, further validations are needed in multiple centers with larger samples in China.

## Introduction

Stroke is the second leading cause of death worldwide with an annual mortality rate of about 5.5 million (1, 2), and the first in China (3). Transient ischemic attack (TIA) is a transient episode of local neurologic dysfunction and it is a prodromal stage of stroke especially in the first few days (4). However, the precise estimate of TIA is difficult mainly due to the varying criteria utilized to identify a TIA. TIA was classically defined as a focal cerebral ischemic event with symptoms lasting <24 h. Due to the rapid development of neuroimaging, up to one-third of patients with TIA may have radiological evidence of acute infarction (5, 6). The frequency of positive diffusion-weighted imaging (DWI) findings varied from 9 to 67% among different cohorts of patients with TIA (7–9). Therefore, the definition of TIA is changing from “time-based TIA” to “tissue-based TIA,” which is “a transient episode of neurological dysfunction caused by focal brain, spinal cord, or retinal ischemia, without acute infarction” (10). Changing the definition of TIA could lead to a landmark to evaluate the progress of stroke.

It has been reported 20% of stroke could be preceded by an episode of TIA. TIA represents really a major warning, and patients with TIA have the highest risk of early, recurrent stroke or other cardiovascular events (11–13). TIA is poorly managed in many countries, thus, it is of great significance to focus on the early differentiation TIA from minor stroke or common TIA mimics (14). As for diagnostic algorithms for TIA, as far as we know, there have been only two tools previously developed. The first clinical scoring tool was Dawson score (Table 1), however, it is less accurate in primary care than in specialist care (16), and lack of retinal and posterior circulation cerebrovascular events assessment (15). The second tool was the Diagnosis of TIA (DOT) score (Table 2), which was considered to be a new tool for non-specialists to make the diagnosis of TIA with greater accuracy with the sensitivity and specificity were 89 and 76%, respectively. The area under the curve (AUC) of DOT score was 0.89, however, the Dawson score was 0.77 (17). However, this new tool of DOT has not yet been externally validated in other populations before widely utilized. Thus, the aim of the present study was aimed to investigate the validation of DOT score as a new clinical diagnostic tool of TIA in patients with transient neurological symptoms.

**Table 1:** TIA scoring system (Dawson score) (15).

**Variable**	**Score if yes**	**Score if no**	**Std▪ error (*P*-value)**
History of stroke or TIA	0.5	0	0.1 (3.5 × 10^−7^)
Headache	0	0.5	0.11 (7.1 × 10^−5^)
Diplopia	1.2	0	0.28 (2.7 × 10-^6^)
Loss of consciousness/Pre-syncope	0	1.1	0.21 (1.9 × 10^−7^)
Seizure	0	1.6	0.43 (1.4 × 10^−4^)
Speech abnormalities	1.3	0	0.14 (<1 10^−10^)
Unilateral limb weakness	1.7	0	0.10 (<1 × 10^−10^)
Upper motor neuron facial weakness	0.6	0	0.15 (9.5 × 10^−8^)
Age	Multiply by 0.04		0.004 (<1 × 10^−10^)

*All values reflect the regression coefficients seen and P-values refer to significance of each variable in the final model. Std. Error, Standard Error. To calculate the score, all values should be summed. If total score >6.1, classify as TIA. For “2:1 cost ratio,” if total score >5.4, classify as TIA*.

**Table 2:** The diagnosis of transient ischemic attack (DOT) score (17).

**Item**	**Input**	**Explanatory notes**
Age	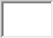	Enter age in years.
History of hypertension	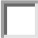	Select if patient has a history of hypertension even if recently diagnosed.
Atrial fibrillation (AF or PAF)	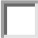	Select if patient has known AF, PAF or atrial flutter or has just been found by you to be in AF.
Dysphasia (disorder of language)	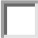	Select ONLY if patient had word finding difficulties, jumbled speech or was unable to speak. Slurring of speech (dysarthria) does NOT count as dysphasia.
Unilateral facial weakness	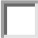	Select if patient had unilateral upper motor neuron (forehead sparing) facial weakness. If patient has isolated facial weakness at present and it is a lower motor neuron weakness, consider Bells Palsy.
Unilateral weakness of arm, leg or both	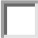	This must be GENUINE weakness. Tingling, numbness, heaviness, deadness or pain does NOT count unless there was true weakness. Ask if it was difficult to move the limb or grip.
Unilateral sensory loss	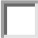	This must be genuine LOSS of sensation. Tingling, numbness or deadness does NOT count unless patient is sure there was loss of pain, temperature or touch sensation.
Visual loss in one eye	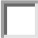	Either partial or complete monocular blindness. Check if patient is sure it was one eye—did they close each eye in turn? Transient loss can be due to a TIA affecting the eye. Persistent visual loss can have a broader differential diagnosis and in all cases, an ophthalmology review is required.
Visual loss in both eyes	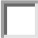	Applies to complete blindness affecting both eyes.
Diplopia	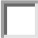	Double vision. Does NOT apply to non-specific blurring of vision.
Homonymous hemianopia	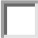	Applies to visual loss in either the right or left visual field. Please do not mistake this for monocular blindness or vice versa.
Visual aura	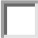	Applies to scintillations (flashing lights), fortification spectra (zig-zag lines) or spreading scotoma as in a migraine type visual aura.
Ataxia	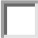	Applies to inco-ordination of the limbs or gait.
Headache	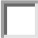	Applies to any headache before, with or after the episode.
Amnesia	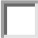	Does the patient remember the episode? Do not select if patient has dementia and is unlikely to remember what happened.
Loss of consciousness or near LOC	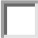	This applies to loss of consciousness due to any reason or near LOC.
Tingling and numbness	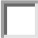	This applies to tingling, numbness or pins and needles to any part of the body including face.
Evaluate	Calculate DOTS	Reset

## Materials and Methods

### Subjects

Our study was a retrospective, observational study in the Department of Neurology, Beijing Chaoyang Hospital, Capital Medical University. We retrospectively enrolled 500 patients with transient neurological symptoms between Jan 2016 and Dec 2018 and they all underwent magnetic resonance imaging (MRI) scans. TIA is classically defined to be the presence of focal neurological symptoms due to a vascular etiology lasting <24 h, irrespective of imaging findings (18). Patients with transient neurological symptoms were divided into two subgroups: TIA mimic group, and cerebrovascular events group including tissue-based TIA (DWI negative) and minor stroke (DWI positive). The tissue-based definition of TIA was proposed to classify patients only if the symptoms fit the clinical syndrome and no acute ischemic lesion identified by DWI (10), whereas minor stroke was similar to the above but positive DWI. However, there are some patients with transient focal neurological symptoms which are not due to focal cerebral ischemia, thus, the patients with an underlying non-ischemic disorder were classified as TIA mimics (such as epileptic seizures, migraine) (19). Our work was approved by the Ethics Committee of Beijing Chaoyang Hospital, Capital Medical University. Written informed consent was obtained from all participants.

### Clinical Variables

We retrospectively obtained the following clinical data: age, gender; vascular risk factors such as hypertension, diabetes mellitus, hyperlipidemia, stroke, coronary heart disease, atrial fibrillation, and current smoking. The laboratory blood tests were also obtained from medical records, including the counts of red blood cell, white blood cell, platelet, hemoglobin, fibrinogen, fasting blood glucose, hemoglobin A1c, glycated albumin, uric acid, homocysteine, total cholesterol, low-density lipoprotein, high-density lipoprotein, triglyceride, C-reactive protein, albumin, pre-albumin, folic acid and 25-hydroxy vitamin D. The scores of Dawson and DOT were also calculated. The presenting symptoms of TIA included motor weakness, sensory disturbance, speech abnormalities (dysarthria or aphasia), loss of consciousness, ataxia, diplopia, and hemianopia. The durations of TIA were classified into three categories: <10 min, 10–59 min, or more than 1 h. The time from symptom onset to MRI and the used medication were also documented.

### Statistical Analysis

The data were described using the mean and standard deviation values for continuous variables, the median and interquartile range values for categorical variables, and absolute numbers and percentages for nominal and categorical variables, and we compared the groups using the nonparametric Mann-Whitney *U*-test. We performed a chi-square test between categorical variables and a *t*-test between continuous variables. The dependent variable was “definite cerebrovascular events,” which included tissue-based TIA and minor stroke (17). The calibration of the models was tested by calibration plots and the Hosmer-Lemeshow statistic, and the discrimination was tested by Receiver Operating Characteristic (ROC) curves and AUC. We used the Statistical Package for Social Sciences (SPSS) version 16.0 (SPSS Inc., Chicago, IL, USA) for data analysis. A *P*-value <0.05 was considered statistically significant.

## Results

We enrolled a total of 500 patients with transient neurological symptoms, and the mean age was 61.1 ± 12.8 years, and 70% (*N* = 350) were male. Among the patients with transient neurological symptoms, we found that TIA mimics (*N* = 140, 28%) and cerebrovascular events including tissue-based TIA (*N* = 252, 50.4%) and minor stroke (*N* = 108, 21.6%).

Table 3 showed the demographics, baseline clinical characteristics, vascular risk factors, the duration of TIA and the time from symptom onset to MRI. In cerebrovascular events group, the use of medication was shown: the aspirin (36.9%), clopidogrel (19.4%), dual antiplatelet therapy (42%), and anticoagulation (1.7%). The laboratory findings were presented in Table 4. We found there were significant differences in diastolic blood pressure, uric acid and homocysteine, the proportion of motor weakness and speech abnormalities, and the scores of Dawson and DOT between the two groups (*P* < 0.05).

**Table 3:** The baseline demographics, clinical characteristics between the two groups.

**Variables**	**TIA mimics (140)**	**Cerebrovascular events (360)**	***P***
**Demographics**			
Age(Y)	60.1 ± 12.0	61.5 ± 13.1	0.273
Sex(Male, %)	90 (64.3%)	260 (72.2%)	0.082
Systolic blood pressure(mmHg)	143.6 ± 20.4	146.9 ± 21.2	0.117
Diastolic blood pressure(mmHg)	79.8 ± 11.6	83.5 ± 13.6	0.003^*^
**Vascular risk factors**			
Hypertension	78 (55.7%)	229 (63.6%)	0.103
Diabetes mellitus	50 (31.2%)	110 (26.5%)	0.267
Coronary disease	8 (5.7%)	28 (7.8%)	0.423
Prior stroke	22 (15.7%)	71 (19.7%)	0.301
Hypercholesterolemia	104 (74.3%)	255 (70.8%)	0.441
Atrial fibrillation	2 (1.4%)	12 (3.3%)	0.246
Peripheral arterial disease	5 (3.6%)	5 (1.4%)	0.118
Smoking	59 (42.1%)	184 (51.1%)	0.072
**Clinical features**			
Motor weakness	48 (34.3%)	231 (64.2%)	0.001^*^
Sensory disturbance	31 (22.1%)	105 (29.2%)	0.113
Speech abnormalities	36 (25.7%)	145 (40.3%)	0.002^*^
Ataxia	7 (5.0%)	19 (5.3%)	0.900
Diplopia	9 (6.4%)	19 (5.3%)	0.615
Loss of consciousness	16 (11.4%)	25 (6.9%)	0.101
Hemianopia	2 (1.4%)	14 (3.9%)	0.160
**Duration of TIA**			0.846
<10 min	60 (45.5%)	150 (47.6%)	
10–59 min	50 (37.9%)	142 (40.3%)	
1 h	22 (16.7%)	60 (17%)	
Time from symptom onset to MRI, days	5 (3-11)	6 (4-13.75)	0.224

*Data were presented as median with interquartile range in parentheses or number with the percentage in parentheses*.

* *P < 0.05*.

*TIA, transient ischemic attack; MRI, magnetic resonance imaging*.

**Table 4:** The laboratory findings and the risk scores of TIA in all TIA patients.

**Variables**	**TIA mimics (140)**	**Cerebrovascular events (360)**	***P***
White blood cell (10^9^/L)	7.1 ± 20	7.0 ± 2.2	0.636
Red blood cell (10^12^/L)	4.5 ± 0.7	4.5 ± 0.6	0.999
Hemoglobin (g/L)	141 ± 14.7	141 ± 17.5	0.989
Platelet (10^9^/L)	213.6 ± 52.4	215 ± 59.6	0.798
Albumin (g/L)	41.9 ± 7.4	42.5 ± 7.2	0.363
Pre-albumin (g/L)	0.3 ± 0.7	0.3 ± 0.5	0.285
Total cholesterol (mmol/L)	4.7 ± 1.0	4.6 ± 1.0	0.239
High-density lipoprotein (mmol/L)	1.1 ± 0.3	1.1 ± 0.4	0.449
Low-density lipoprotein (mmol/L)	2.8 ± 0.8	2.7 ± 0.9	0.07
Triglyceride (mmol/L)	1.8 ± 1.5	2.0 ± 2.0	0.296
Uric acid (μmol/L)	312.2 ± 89.1	337.7 ± 88	0.004^*^
Homocysteine (μmol/L)	15.1 ± 7.0	17.8 ± 10.3	0.007^*^
Folic acid(ng/ml)	6.7 ± 5.1	6.1 ± 4.8	0.251
25-hydroxy vitamin D (ng/mL)	14.5 ± 4.8	15.2 ± 12.7	0.752
C-reactive protein (mg/L)	1.6 (0.8~2.7)	1.7 (0.8~3.0)	0.404
Fibrinogen (mg/dl)	267.3 ± 66.6	272.9 ± 78.7	0.468
Fasting blood glucose (mmol/L)	6.4 ± 2.3	6.8 ± 3.3	0.209
hemoglobin A1c (%)	6.3 ± 1.1	6.4 ± 1.4	0.880
Glycated albumin(%)	13.7 ± 3.6	15.1 ± 4.1	0.398
Dawson score	6.5 ± 1.3	7.3 ± 1.4	0.001^*^
DOT score	−0.04 (−2.1~0.92)	1.25 (0.31~3.47)	0.001^*^

*Values represent number (percent) for categorical variables and mean ± SD or median (interquartile range) for continuous variables*.

* *P < 0.05*.

*DOT, diagnosis of TIA*.

**Figure 1:**
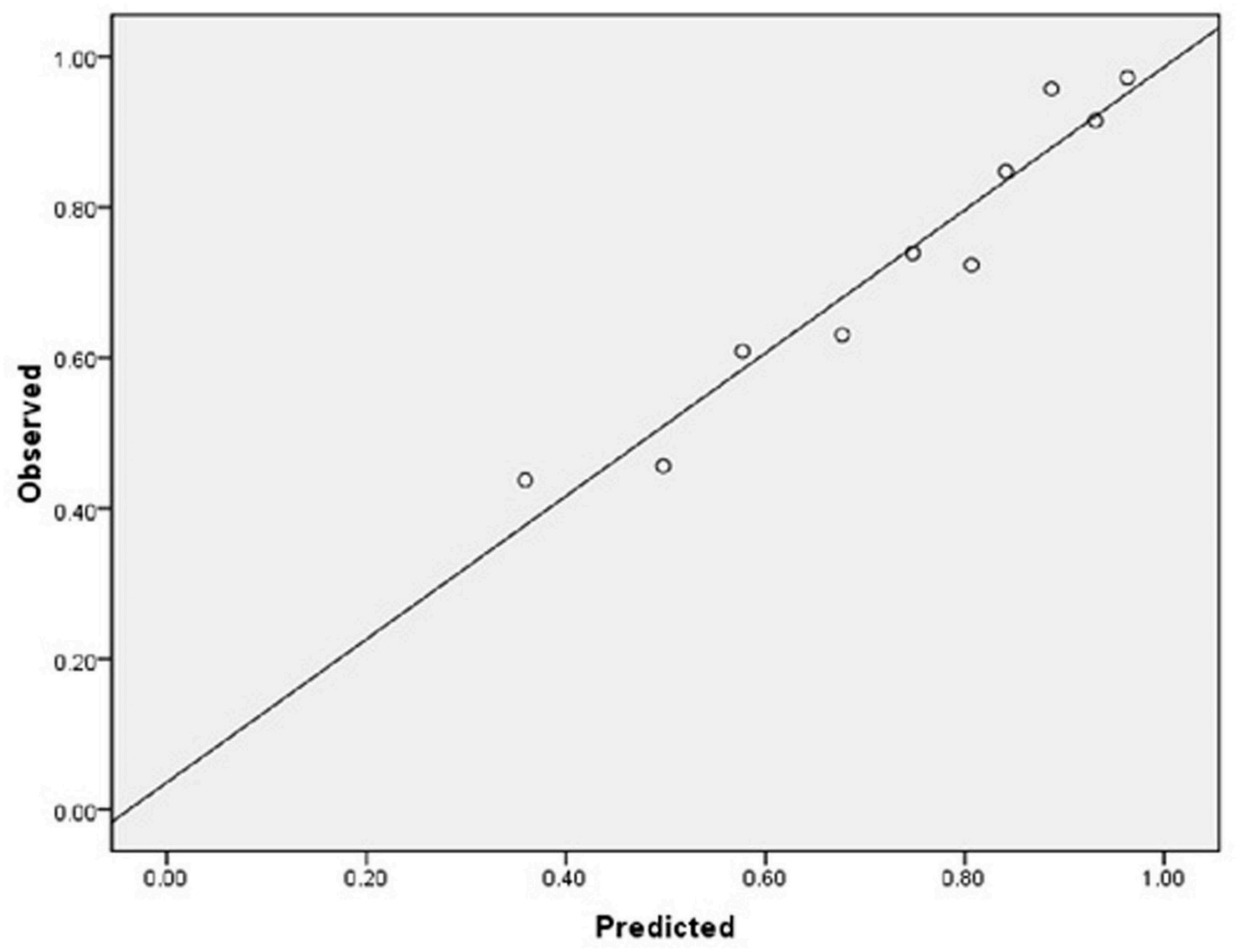
The calibration plots (predicted vs. observed results).

**Figure 2:**
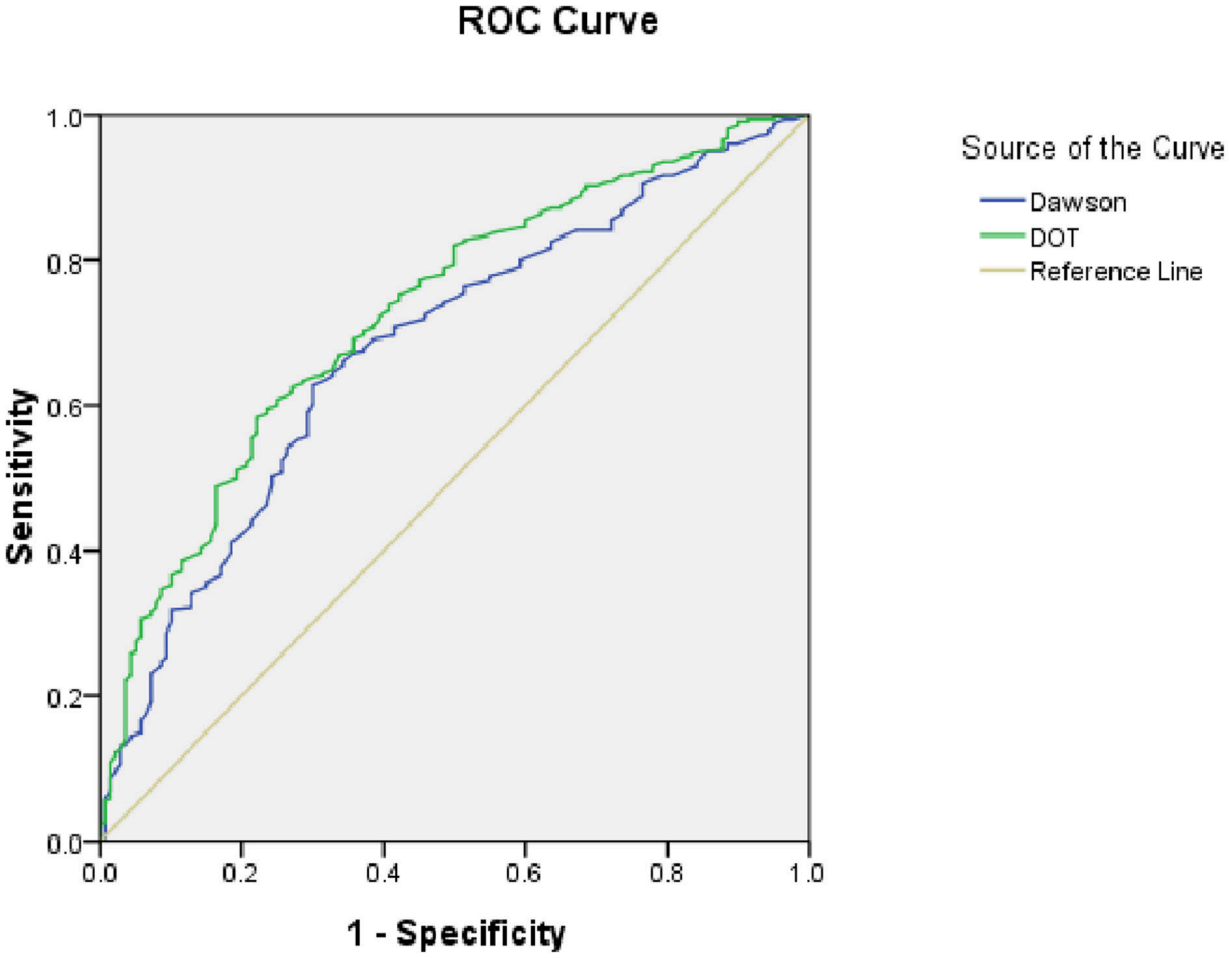
The predictive value of Dawson and DOT score using ROC.

As for the calibration and discrimination, predicted and observed diagnoses were plotted by the AUC with 95% *CI*. Figure 1 showed the calibration plots (predicted vs. observed results), and the *P-*value was 0.541 for the Hosmer-Lemeshow test of goodness of fit. From Table 5 and Figure 2, we found the AUC (95% *CI)* of the Dawson and DOT for predicting the cerebrovascular events were 0.681 (95% *CI*, 0.629–0.732) and 0.728 (95% *CI*, 0.680–0.776), respectively. The score of DOT showed a greater AUC, with sensitivity of 70.3% and specificity of 62.9%, respectively. The optimal cut point for DOT score was 0.455 using the Youden Index.

**Table 5:** The area under the curve of the scores of TIA.

**Variable(s)**	**Area under the curve**	***P***	**95% Confidence interval**	
Dawson	0.681	0.001	0.629	0.732
DOT	0.728	0.001	0.680	0.776

## Discussion

In our present study, among patients with transient neurological symptoms, the patients with cerebrovascular events were more likely to be higher diastolic blood pressure, uric acid and homocysteine, more motor weakness and speech abnormalities, and higher scores of Dawson and DOT. DOT score showed relatively good calibration and discrimination to identify of TIA in a Chinese population.

TIA requires urgent investigation and treatment. The term “transient neurological symptoms” is used to describe a broad spectrum of symptoms following TIA or TIA mimics (20). Unilateral weakness and speech disturbances are the most common clinical manifestations of TIA, and our findings were in accordance with the prior studies (21, 22). It was reported unilateral weakness and speech disturbance were found in ~31–54% and 25–42% of TIA, respectively (5). There is no gold standard clinical tool used to diagnose TIA or stroke only based on symptomology, thus, accurate identification of TIA or stroke patients is quite difficult. It was reported up to 50–60% of TIA patients were diagnosed to be non-cerebrovascular mimics by non-specialists (23). Another data showed that up to 35% of referrals to TIA clinics were non-vascular mimics (24). Thus, it is especially crucial about the differential diagnosis of transient neurological symptoms from TIA or TIA mimics in clinical practice (e.g., migraine or seizure) (20, 25).

As a result, a TIA diagnostic tool to differentiate some TIA mimics from urgent TIA would be much valuable. To date, there have been only two tools for the early diagnosis of TIA. The clinical scoring system of Dawson was proved to facilitate to detect of TIA accurately. However, it has some limitations both on the utility only in primary care and not including posterior circulation cerebrovascular events (15). The second tool was the DOT score, which was recognized to be a new tool to make the diagnosis of TIA more accurate (17). However, this new tool of DOT has not yet been externally validated in other populations out of the United Kingdom. Our findings indicated that DOT score performed relatively good calibration and discrimination, with a sensitivity of 70.3% and specificity of 62.9%. We also found there was greater AUC of DOT (0.728) compared with Dawson (0.681). According to Dutta D study in 2016, the AUC for DOT was 0.89 and Dawson score was 0.77. Our findings were consistent with Dutta D study (17).

Our study also had some limitations. First, this study was designed as a retrospective study from a single center, which is one potential limitation. Studies with larger numbers from multiple centers in China are needed to confirm our findings. Second, we did not perform follow-ups to assess the risk factors of the recurrence of TIA or stroke, which may be of great importance for the assessment and management of TIA. Third, we did not perform the etiological classification of stroke/TIA, according to the Trial of Org 10172 in Acute Stroke Treatment. Fourth, we did not find blood or imaging biomarkers available to reliably distinguish TIA from TIA mimics (22).

Despite these limitations, as far as we know, the present study is the largest ever-reported study to identify the clinical predictors for the accurate diagnosis of TIA. Besides, for the first time, we have externally validated the DOT score as for a new tool of TIA in patients with TIA in China. Thirdly, we further confirmed the score of DOT could encompass the entire spectrum of TIA/stroke, which could be used as a mobile app or web-based calculator with higher accuracy.

In summary, we have externally validated the DOT score in patients with TIA in a Chinese population, and it was proved to be accurate as a diagnostic tool for TIA in China. The relative merits and clinical utility of the DOT score will warrant further prospective study in multiple centers with large samples.

## Data Availability

The datasets for this manuscript are not publicly available because still recruit and in progress. Requests to access the datasets should be directed to JY, yuan_doctor@163.com.

## Ethics Statement

Our work was approved by the Ethics Committee of Beijing Chaoyang Hospital, Capital Medical University. Written informed consent was obtained from all participants.

## Author Contributions

JY, ZJ, and YS examined, evaluated the patient. JY drafted and revised the manuscript. WH participated in the design of the case-report and helped to draft the manuscript. All authors read and approved the final manuscript.

### Conflict of Interest Statement

The authors declare that the research was conducted in the absence of any commercial or financial relationships that could be construed as a potential conflict of interest.
